# Habitat Fragmentation and Ecological Traits Influence the Prevalence of Avian Blood Parasites in a Tropical Rainforest Landscape

**DOI:** 10.1371/journal.pone.0076227

**Published:** 2013-10-04

**Authors:** Susan G. W. Laurance, Dean Jones, David Westcott, Adam Mckeown, Graham Harrington, David W. Hilbert

**Affiliations:** 1 Centre for Tropical Environmental and Sustainability Studies (TESS), James Cook University, Cairns, Queensland, Australia; 2 School of Marine and Tropical Biology, James Cook University, Cairns, Queensland, Australia; 3 Division of Ecosystem Sciences, The Commonwealth Scientific and Industrial Research Organisation, Tropical Forest Research Centre, Atherton, Queensland, Australia; Universidade Federal de Minas Gerais, Brazil

## Abstract

In the tropical rainforests of northern Australia, we investigated the effects of habitat fragmentation and ecological parameters on the prevalence of blood-borne parasites (*Plasmodium* and *Haemoproteus*) in bird communities. Using mist-nets on forest edges and interiors, we sampled bird communities across six study sites: 3 large fragments (20–85 ha) and 3 continuous-forest sites. From 335 mist-net captures, we recorded 28 bird species and screened 299 bird samples with PCR to amplify and detect target DNA. Of the 28 bird species sampled, 19 were infected with *Plasmodium* and/or *Haemoproteus* and 9 species were without infection. Over one third of screened birds (99 individuals) were positive for *Haemoproteus* and/or *Plasmodium*. In forest fragments, bird capture rates were significantly higher than in continuous forests, but bird species richness did not differ. Unexpectedly, we found that the prevalence of the dominant haemosporidian infection, *Haemoproteus*, was significantly higher in continuous forest than in habitat fragments. Further, we found that ecological traits such as diet, foraging height, habitat specialisation and distributional ranges were significantly associated with blood-borne infections.

## Introduction

Emerging infectious diseases are a growing global concern and could have large impacts on wildlife and human populations [1], [2]. By definition, such diseases have recently moved to a new host, increased in prevalence or range, are newly discovered, or are recently evolved [3]–[5]. Anthropogenic land-use change is considered a key driver of disease emergence because it can result in novel interactions among vectors, hosts and diseases [6]. In tropical regions, emerging pathogens have been associated with a range of land-use activities including deforestation, habitat fragmentation, urbanization, bushmeat hunting, gold mining and road construction [4], [7].

Habitat fragmentation is a ubiquitous feature of human land use and can cause major changes in the structure, function and composition of tropical rainforest communities [8], [9]. Fragmentation has been hypothesized to alter disease dynamics and facilitate the rise of infectious diseases in at least four ways. First, forest edges dramatically increase in fragmented landscapes [10]. These rapidly expanding edge environments juxtapose dense rainforest with open pasture or cropland and provide opportunities for vectors or hosts to move between habitats, increasing their potential disease exposure through novel species interactions [11], [12]. Second, the replacement of rainforests with anthropogenic lands has been shown dramatically to increase the abundance of open-habitat preferring mosquitoes such as *Anopheles*, thereby elevating the probability of disease transmission via an increase in vectors [13]–[15]. Third, rodents, which can be important reservoirs for diseases such as hantavirus [16], frequently increase in abundance in temperate [17] and tropical [18], [19] forest fragments. Finally, by diminishing vertebrate-host diversity [20], [21], habitat fragmentation can increase pathogen prevalence because vectors are concentrated on the remaining species and thereby increase their disease load [22].

Predicting the influence of fragmentation on parasite incidence is important but challenging because there is so little known about many wildlife diseases. As a result, research tends to focus on well-known disease models in order to understand the mechanisms associated with fragmentation and parasite dynamics, such as recent research on habitat fragmentation and avian blood parasites in west Africa [23], [24]. Avian blood parasites, particularly the closely-related genera *Plasmodium* and *Haemoproteus*, make an excellent disease model as they have been studied since the 19^th^ century [25], [26]. These widespread parasites infect a range of vertebrate hosts and blood-sucking insects, but have different transmission cycles and pathogenicity. Culicine mosquitoes are the primary vectors of *Plasmodium* spp., and hippoboscid flies and ceratopogonid midges the vectors of *Haemoproteus* spp. [25], [27].

The pathogenicity of *Haemoproteus* and *Plasmodium* parasites in birds is highly variable; both genera can destroy blood cells and cause anemia, with *Plasmodium* generally having the more severe pathology [25], [28]. Recent experimental approaches, which reduce avian infections through medication, have found that *Plasmodium*, *Haemoproteus* and *Leucoytozoan* infections can significantly lower the reproductive performance of wild birds [29]–[31]. Furthermore, when *Haemoproteus* infections were controlled in blue tits (*Cyanistes caerules*), female survival was significantly enhanced [32]. These results indicate that chronic parasite infections can play a significant role in bird fitness [31].

The prevalence and intensity of blood-parasite infections in birds have also been associated with a number of biological and ecological characteristics [33]. For example, biological parameters such as sex, age, plumage colour, embryonic development, and body condition have all been correlated with increased infections [33]–[35]. Furthermore, parasitological studies have found that habitat selection, elevational distributions, diet, nest type and participation in colonies or mixed-species flocks [36]–[39] can influence infection rates. These factors may play a pivotal role in host or vector-community structure, which in turn can influence parasite transmission.

We investigated the effects of habitat fragmentation on the prevalence of *Plasmodium* and *Haemoproteus* blood parasites in rainforest birds in northeastern Australia. We focus here on three questions: (1) Does pathogen prevalence differ between bird communities in fragmented and continuous forest? (2) Are ecological and biological traits of species associated with parasite prevalence? (3) Is parasite prevalence influenced by bird abundance? We hypothesise that habitat fragments will be under-resourced, supporting smaller bird populations with higher disease prevalence because of lower host dilution. Further, following from earlier studies [37], [38], we hypothesise that common, terrestrial insectivores restricted to rainforest habitats will have higher disease prevalence than do rarer, wide-ranging generalists.

## Materials and Methods

### Ethical Statement

The study was carried out with animal handling permits issued from the Queensland Department of Environment and Heritage Protection (WIT04758807) and the Australian Bird and Bat Banding Scheme (SL 2960 & GH 662). Animal handling ethics approval was provided by James Cook University (#A1591) and CSIRO (#07-02). Bird handling followed strict hygiene requirements and short capture times to minimise individual animal stress. All captured individuals were released.

### Study site and bird sampling

**Figure 1:**
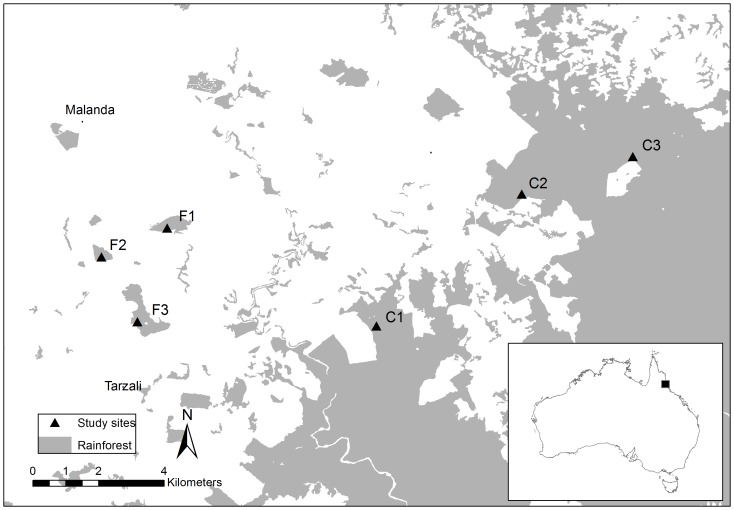
The distribution of fragmented (F1–F3) and continuous rainforest (C1–C3) study sites on the Atherton Tablelands in north-eastern Australia.

The study was undertaken on the Atherton tableland, a mid-elevation plateau (600–900 m) in northeastern Australia. The region experiences a mild tropical climate with annual rainfall of 2500 mm/yr and monthly temperatures from 16 – 29°C. Our six study sites support tropical rainforest classified structurally as complex mesophyll vine forest [40] and were chosen to control for elevation, soil type and rainfall. Three habitat fragments were selected, of 20, 39 and 85 ha in area; all occur within a 4-km radius and have been isolated for ∼50 years. Three-continuous forest sites (part of a ∼100 000 ha forest tract) were established 7–15 km from the fragments to the northeast and southeast (Fig.1).

At each of the six sites, bird abundance data were estimated using mark-recapture methods with nylon mist-nets established along two parallel 100 m transects. One transect was established along the forest edge inside the treeline, with a second transect in the forest interior (>100 m from the nearest edge). Each site was netted for two consecutive days from 6–18 November 2011, with all nets opened from 0600 to 1400 hrs. Nets were checked at 15–20 minute intervals with captured birds identified, weighed, measured, aged, breeding status identified and given a uniquely numbered bird band. A small sample of peripheral blood was collected from the brachial vein of captured birds and preserved on Whatman FTA Cards (Whatman International Ltd. UK). This study was undertaken at the commencement of the bird breeding season.

### Parasite detection

We detected *Plasmodium* and *Haemoproteus* parasites using a PCR technique devised by Beadell and Fleischer (2005). This ‘restriction enzyme-based diagnostic assay’ is a reliable, inexpensive method that allows for the initial characterization of avian blood parasites to genera [41]. DNA was extracted from whole blood using QIAGENS's BioSprint 15, Dried Blood Spots protocol. We used primers 213F and 372R and methods (outlined in Beadell and Fleischer 2005) to amplify a 160 bp *Xmn*I site unique to *Haemoproteus*. We ran our amplifications as follows: 95 C for 5 min, 92 for 30 sec, then a touchdown from 57 to 53 C at 1 degree C intervals for 30 seconds, then 30 cycles at 52 C. Each cycle had a 72 C, 30 second extension and a final extension at 72 C for 7 minutes. A restriction enzyme XmnI was then added to the PCR product. Any product from *Haemoproteus* were cleaved into fragments of 121 and 39 bp while *Plasmodium* spp remained undigested at 160 bp. Multiple-genera infections were also resolved by visualising the different sized PCR products on a 4% agarose gel.

### Statistical analyses

Habitat fragmentation effects on bird captures, species richness and disease prevalence was assessed using two-way ANOVAs for all species pooled. We contrasted (1) fragments vs. continuous forest, (2) forest edge vs. interior sites, and tested for statistical interactions. We evaluated gradients in the bird community composition between fragments and continuous forest with nonmetric multidimensional scaling ordination (NMS) in the PC-ORD Package [42]. Data were log-transformed prior to analysis and Monte-Carlo randomizations (100 runs) were undertaken to determine if ordination axes explained significantly more variation than expected by chance. We tested the null hypothesis that there was no difference between bird community composition in fragmented and intact tropical rainforests, and on forest edges and interiors, with a two-way Permutation Multivariate Analysis of Variance (PerMANOVA; [43]). Individual species responses to fragmentation and parasite prevalence could not be explored due to limited sample sizes for many species. However, we examined associations between species with similar ecological attributes and their frequency of blood-parasite infections using Chi-square tests.

Four ecological parameters of birds were studied: diet, foraging height, rainforest specialization and distributional range. Bird species were categorised *a priori* from published life-history and distributional data [44]–[46]. We categorised birds into three dietary functional groups: *insectivore, frugivore/nectarivore*, and *omnivore* based on predominant food choice. Bird species were identified as using one of four foraging-height classes: *terrestrial*, *understorey*, *midstorey* and *canopy*. Habitat use was classified as one of the following: *rainforest* (only), *closed forests* (which include rainforests, eucalypt forests and mangroves), and *all habitats*, including natural and human-altered habitats in Australia. Four categories for distributional range included *very restricted* (endemic species restricted to the Wet Tropics biogeographic region; *restricted* (species restricted to rainforests from two biogeographic regions such as Wet Tropics and Cape York or south-east Queensland); *moderate ranging* (species found along the eastern coast of Australia); and *wide ranging* (species found throughout Australia). Our findings were tested against the published results of an independent study undertaken in the same region [47].

## Results

From 335 mist-net captures, we recorded 28 rainforest bird species from thirteen families. We screened 299 individuals for blood parasites and found that 99 birds (33.1%) were positive for either *Plasmodium* and/or *Haemoproteus* infections. *Haemoproteus* dominated the infections, occurring in 83 individuals (27.8%), with *Plasmodium* present in 38 birds (12.7%). Of the 28 bird species examined, 19 had detectable parasitic infections of *Plasmodium* or *Haemoproteus*, and 9 were free from infection. Although 11 species were determined to carry either parasite genera, 9 species were found to carry both simultaneously (Table 1).

**Table 1:** Total captures and blood-parasite infections of rainforest bird species in fragmented and intact habitats of the Atherton Tablelands, north-eastern Australia.

FAMILY/Species	Captures	*Haemoproteus*	*Plasmodium*	Multiple
**ACATHIZIDAE**				
*Gerygone mouki*	2	0	0	0
*Gerygone palpebrosa*	1	0	0	0
*Oreoscopus gutturalis*	2	0	0	0
*Sericornis citreogularis*	22	1	1	1
*Sericornis keri*	27	4	2	1
*Sericornis magnirostra*	39	3	1	1
**CINCLOSOMATIDAE**				
*Psophodes olivaceus*	2	0	0	0
**CLIMACTERIDAE**				
*Cormobates leucophaea*	5	0	1	0
**COLUMBIDAE**				
*Chalcophaps indica*	1	0	0	0
**MELIPHAGIDAE**				
*Acanthorhynchus tenuirostris*	3	1	0	0
*Lichenistomus frenatus*	1	1	1	1
*Meliphaga lewinii*	12	0	0	0
*Xanthotis macleayanus*	3	2	1	1
**MONARCHIDAE**				
*Arses kaupi*	1	0	0	0
*Machaerirhynchus flaviventer*	1	1	0	0
*Monarcha melanopsis*	15	5	0	0
*Monarcha trivirgatus*	22	2	4	0
**ORTHONYCHIDAE**				
*Orthonyx spaldingii*	1	1	0	0
**PACHYCEPHALIDAE**				
*Colluricincla boweri*	27	7	4	2
*Colluricincla megarhyncha*	15	0	0	0
*Pachycephala pectoralis*	10	1	1	0
**PARADISAEIDEAE**				
*Ptiloris victoriae*	5	1	1	0
**PETROICIDAE**				
*Heteromyias cinereifrons*	58	48	14	13
*Tregellasia capito*	3	2	0	0
**PTILONORHYNCHIDAE**				
*Ailuroedus melanotis*	6	0	5	0
**RHIPIDURUDAE**				
*Rhipidura albiscapa*	4	0	0	0
*Rhipidura rufifrons*	10	2	1	1
**ZOSTEROPIDAE**				
*Zosterops lateralis*	1	1	1	1

**Figure 2:**
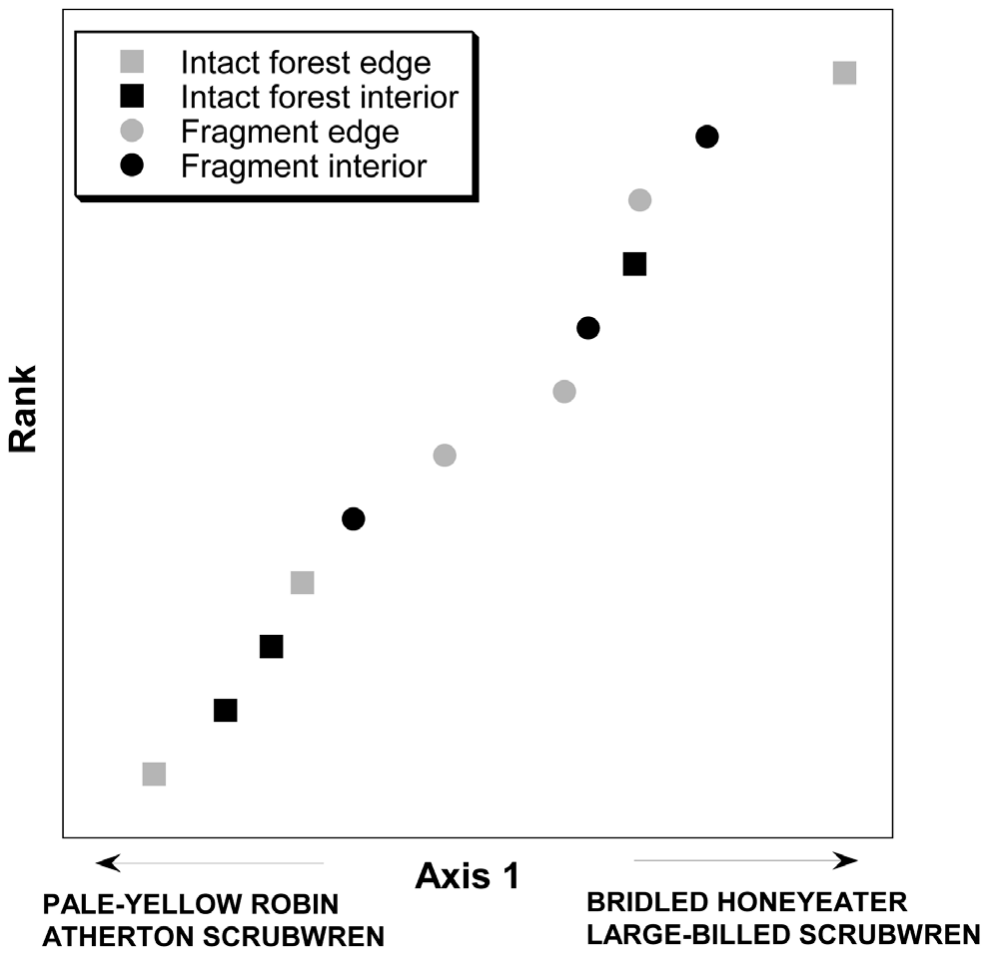
An ordination of bird community composition from forest edges and interiors in rainforest fragments and in intact forest study sites on the Atherton Tablelands, north-eastern Australia. Habitat fragmentation or edge effects did not influence bird community composition at these sites, as illustrated by the single significant ordination axis (explaining 60% of the variance in the dataset).

Based on our short-term sampling, we captured 20% more birds on average in forest fragments (186 individuals) than in continuous forest (149 individuals), a significant result (*F*_1,11_ = 5.44 *P* = 0.044). Fragments tended to have higher species richness compared to continuous forest, a result approaching statistical significance (*F*_1,11_ = 4.61, *P* = 0.064; two-way ANOVA). However, the ordination analysis revealed no marked differences in species composition between fragmented and intact rainforests (Fig. 2). Our ordination analysis identified a significant gradient in the dataset (Axis 1) that explained 59.5% of the data variance, but did not reflect a fragmentation or edge effect pattern in bird community composition (PerMANOVA analysis:Table 2, *P* = 0.227), but was influenced by the distribution of four rainforest species: Large-billed Scrubwren (*Sericornis magnirostra*), Atherton Scrubwren (*Sericornis keri*), Bridled Honeyeater (*Lichenostomus frenatus*) and Pale-yellow Robin (*Tregellasia capito*).

**Table 2:** Two-way PerMANOVA testing whether bird communities (28 species) differ between forest edges and interiors in habitat fragments and intact-forest sites.

SOURCE	d.f.	SS	MS	Pseudo-*F*	*P*
Fragment	1	0.1984	0.1984	1.3834	0.227
Edge	1	0.1479	0.1479	1.0312	0.399
Interaction	1	0.0307	0.0307	0.2143	0.975
Residual	8	1.1476	0.1434		
TOTAL	11	1.5248			

**Figure 3:**
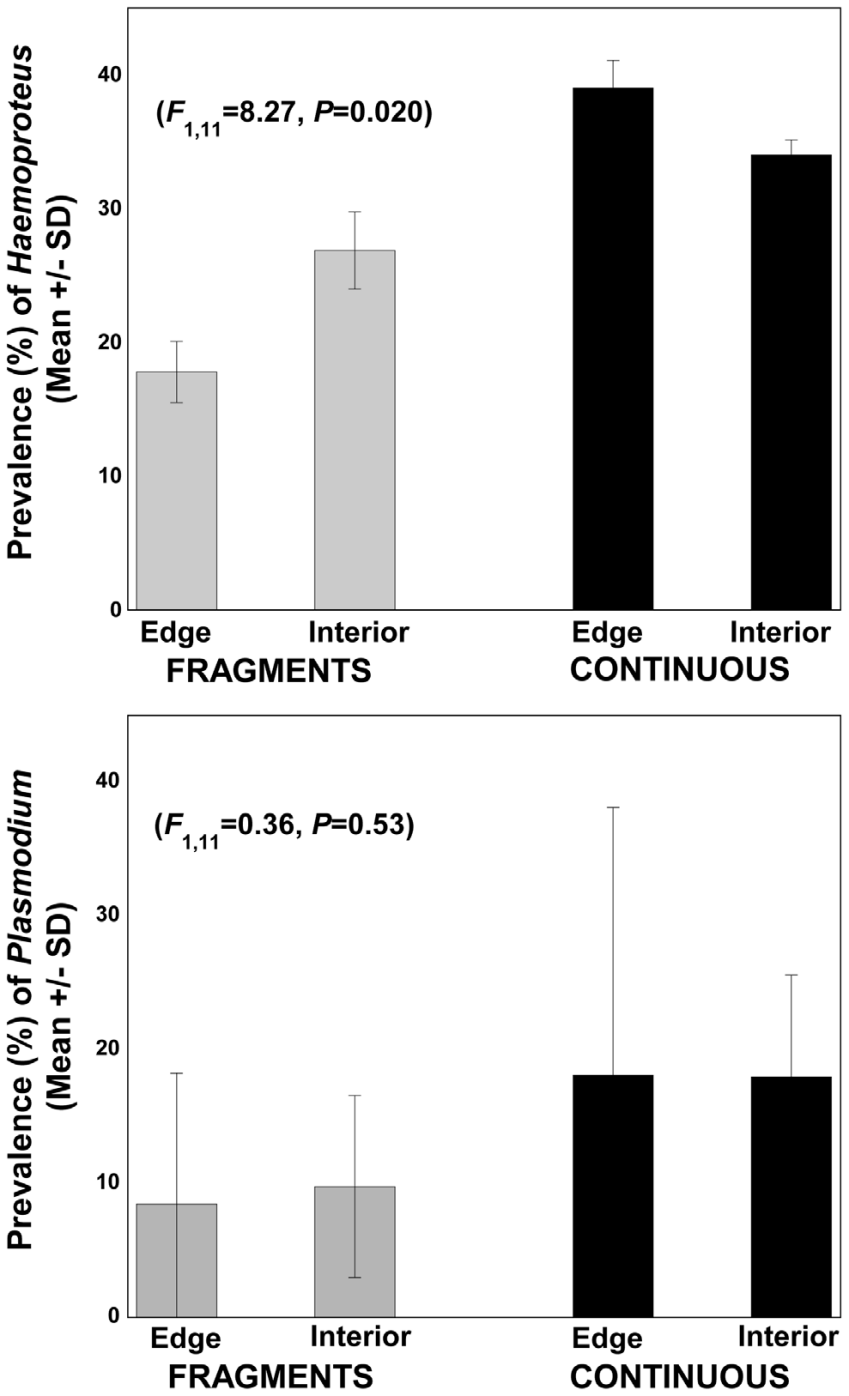
Habitat fragmentation reduced the prevalence of *Haemoproteus* (3A) and *Plasmodium* infections (3B) tropical rainforest of northern Australia.

Unexpectedly, birds in continuous-forest sites had significantly higher prevalence of blood parasites compared to those in forest fragments (*F*_1,11_ = 6.16, *P* = 0.038), but there was no effect of forest edge (*F*_1,11_ = 0.87 *P* = 0.38) or an edge-fragment interaction (*F_1,11_* = 0.61, *P* = 0.46; two-way ANOVA). This pattern was influenced mostly by *Haemoproteus* infections, which were significantly more frequent in continuous forest than fragments (*F*_1,11_ = 8.27, *P* = 0.021; Fig. 3A), with no effect of forest edge (*F*_1,11_ = 0.78 *P* = 0.40) or edge-fragment interaction (*F*_1,11_ = 2.50, *P* = 0.15). *Plasmodium* infections did not differ between continuous forest and fragments (*F*_1,11_ =  0.36, *P* = 0.53; Fig. 3B), and we detected no effect of forest edges (*F*_1,11_ = 1.13, *P* = 0.32) or their interaction (*F*_1,11_ = 0.09, *P* = 0.77). Similarly, multiple infections did not differ between continuous forest or fragments (*F*_1,11_ = 1.07, *P* = 0.33), increase near forest edges (*F*_1,11_ = 0.23, *P* = 0.64) or show evidence any statistical interaction (*F_1,11_* = 0.88, *P* = 0.37; all two-way ANOVAs).

**Figure 4:**
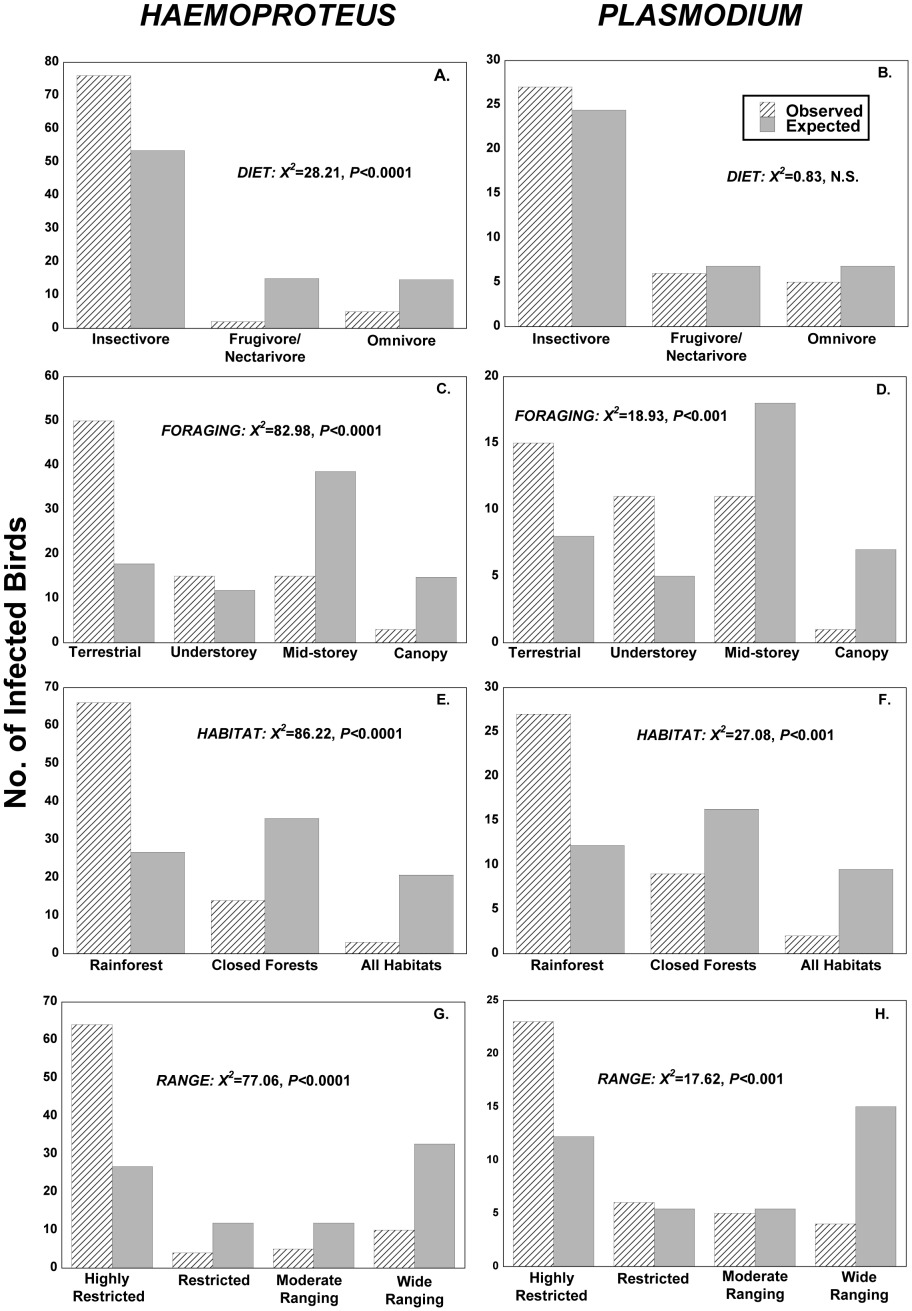
Associations between blood-parasite infections and the ecological traits of birds, assessed using Chi-square tests.

We found a strong effect of ecological traits on *Haemoproteus* and *Plasmodium* infection (Fig. 4). Species with *Haemoproteus* infections were more likely to be insectivorous (Fig. 4A, *P*<0.0001), terrestrial (Fig. 4C, *P*<0.0001), rainforest specialists (Fig. 4E, *P*<0.0001) and highly restricted (endemic) in their distributional range (Fig. 4G, *P*<0.0001; all Chi-Square tests). Similarly, *Plasmodium* infections were associated with terrestrial and understorey species (Fig. 4D, *P*<0.001) and rainforest specialists (Fig. 4F, *P*<0.001) that were highly restricted in range (Fig. 4H, *P*<0.001). *Plasmodium* infections were not associated with dietary preference of bird species (Fig. 4B).

Finally, we found no relationship between the abundance of the 14 most frequently captured species (≥5 individuals) and their prevalence of *Haemoproteus* (*r_s_* = 0.373, *P* = 0.19) or *Plasmodium* (*r_s_* = −0.234, *P* = 0.42) infection (Spearman rank tests).

## Discussion

In tropical Australia, bird communities in continuous rainforest had a significantly higher prevalence of *Haemoproteus* infections than did those in nearby fragmented rainforests. *Plasmodium* infections were less frequent overall, but showed a similar trend of increased prevalence in continuous forest. This result does not appear to be associated with the abundance of bird hosts, which were significantly higher (based on mist-net capture frequency) in forest fragments. We also found no direct relationship between the estimated abundance of bird species and blood-parasite prevalence.

An increase in rainforest area, such as occurs in bigger fragments or continuous forest, has been associated with higher prevalence of *Plasmodium* infections in birds in Cameroon [23], [24] and Brazil [37], and with higher *Haemoproteus* infections in Cameroon [23]. Prior to our study, there had been no comparable investigation of habitat fragmentation and parasite prevalence in the Asia-Pacific region. However, we did not discriminate among specific parasite lineages in our study, and this probably obscured some nuances of host-parasite dynamics [48]. For instance, Chasar et al. (2009) found that, within two bird hosts in Cameroon, infections of *Plasmodium* and *Haemoproteus* morphospecies changed in apparent response to habitat fragmentation.

Nonetheless, our study illustrates some of the complexity of interactions between host-parasite dynamics and landscape ecology. We predicted that parasite prevalence would increase in forest fragments based on our understanding of how fragmentation can (1) lower host diversity and its “dilution” potential on disease prevalence [22], (2) increase vector abundance in adjacent pastures [14], and (3) influence vector-host interactions and host stress [17], [49]. We found, to the contrary, that parasite prevalence was lower in fragments than in nearby intact forest. Large habitat areas are generally associated with more stable and interconnected populations, and our findings suggest that maintaining a stable disease reservoir and vector population might be important for parasite persistence. Further, tropical forest fragments can be hyper-dynamic, with a high turnover of bird and other animal and plant populations [50], [51]. Such instability in fragmented host communities might reduce parasite prevalence.

Fragmentation could potentially influence the mosquito and biting-fly vectors that transmit avian blood parasites. Habitat fragments are generally a non-random sample of the landscape, often associated with steep, rocky, infertile soils or riparian areas, which might support different vector communities than those in continuous forest. Further, microhabitat and environmental conditions are often altered in fragments [52]. For example, large canopy trees that support tree hollows and abundant epiphytic plants are rare in fragments because large trees suffer high mortality rates in fragments [53]. Furthermore, fragments and forest edges suffer increased desiccation [54], that may further influence vector communities by reducing breeding habitats. These features might result in a diminished or less stable vector community in fragments.

Interestingly, we found that several ecological characteristics of birds appeared to influence the incidence of blood-parasite infections. We corroborated our findings by analysing published data from another study of avian blood parasites in our region [47], one that recorded most (24 of 28 species) of the same species we captured. For *Haemoproteus* infections, the study was remarkably similar to ours: infection was significantly higher in bird species that were insectivorous (χ^2^ = 28.21, *P*<0.0001), terrestrial (χ^2^ = 17.62, *P*<0.001), rainforest specialists (χ^2^ = 71.63, *P*<0.0001) and highly restricted in geographic range (χ^2^ = 31.58, *P*<0.0001; all Chi-square tests for independence). However, *Plasmodium* infections were too rare in the second study (n = 4) to compare with our findings.

Our findings suggest there is large interspecific variation in parasite infection rates of rainforest birds. One possible explanation is that avian blood parasites may tend to be host specific. Globally, host conservatism has been found in many *Haemoproteus* and *Plasmodium* lineages [55]. In Australia and Papua New Guinea, for instance, an analysis of 60 *Haemoproteus* and 18 *Plasmodium* strains from 77 bird species found *Haemoproteus* to show marked host-specificity whereas *Plasmodium* did not [56]. In addition, host characteristics such as sex, age, plumage colour, embryonic development, and body condition can potentially influence parasite incidence [33]–[35].

We found that bird species that are rainforest endemics were more likely to carry both *Haemoproteus* and *Plasmodium* infections than did species that are wide-ranging habitat generalists. This may parallel a recent study in Africa, which found that rainforest birds with restricted ranges were more likely to be infected by avian malaria from generalist parasite lineages, whereas wide-ranging species tended to be infected by specialist parasites [38]. We speculate that restricted-endemic species invest less in infection control because of a longer evolutionary association with their blood parasites, which has resulted in reduced parasite virulence. Terrestrial birds were also more likely to be infected and it is possible that these species encounter vectors more frequently than do species associated with higher forest strata, which tend to be harsher environments with hotter, drier and windier conditions than occurs in the forest understorey. In future studies, a focus on specific parasite lineages would likely reveal further nuances of the seemingly complex host-parasite dynamics in fragmented landscapes.
